# 

**Published:** 1897-08-21

**Authors:** 


### EARLY EDITION.

**Notices of Births, Deaths, and Marriages** are inserted in this column at a charge of 2s. 6d. for an announcement not exceeding four lines.

### NOTICE TO CORRESPONDENTS.

All MS., Letters, Books for Review, and other matters intended for the Editor should be addressed The Editor, "The Hospital," The Hospital Building, 28 & 29, Southampton Street, Strand, W.C.

The Editor cannot undertake to return rejected MS., even when accompanied by stamped directed envelope.

**Notice to Advertisers and Subscribers.**

All Advertisements, Orders for Copies of The Hospital, and Business Communications should be addressed to **THE MANAGER, (Not to The Editor),** The Hospital, The Hospital Building, 28 & 29, Southampton Street, Strand, London, W.C.

The Cost of Subscription, post free, is as follows:—Per week, 2½d.; per quarter, 2s. 8d.; per half-year, 5s. 3d.; per annum, 10s. 6d. Foreign:—Per annum, 15s. 2d.; payable, in all cases, in advance.

**NOTICE.—Vol. XXI. of "The Hospital" (October, 1896, to March, 1897), in handsome cloth binding, is on sale at the Office of this Journal, price 7s. 6d. Cases for binding the Numbers contained in this Volume, 1s. 6d. Index to Vol. XXI., 2½d., post free.**

**The Monthly Part for September will be ready on September 1st. Price 6d., by post 8d.**

### BOOKS RECEIVED.

Smith, Elder, and Co.

"Nervous Affections of the Hand." By George Vivian Poore, M.D.

Isbister and Co.

"Wells Cathedral." By the Rev. C. M. Church, M.A. Illustrated by H. Railton.

Baillière, Tindall, and Cox.

"The Physiology of Pregnancy and Child-birth." By Arthur E. Giles, M.D.

Macmillan and Co.

"A System of Medicine," Vol. II. By Many Writers.

B. Konegen.

"Reich's Medicinal Anzeigers," Erklärung des Stotterns dessen Heilung und Verhütung. By Ferdinand Gruenbaum.

Dr. G. Beck's "Therapeutischer Almanach."

"Jahresbericht über die Fortschritte der Diagnostik." By Dr. E. Schill.

P. S. King and Son.

"Pauper Children." Written and Signed on behalf of the Central Committee of Poor Law Conferences, and of the Metropolitan Association for Befriending Young Servants.

Potter and Clarke.

"Etidorhpa; or, The End of the Earth." By Llewellyn Drury.

Kegan Paul, Trench, Trübner, and Co.

"The Book of Tephi." By J. A. Goodchild.

**Periodicals Received.**—Westminster Review, Windsor Magazine, London, Local Government Journal, Medical Record, Industries and Iron, Electrical Review, Guy's Hospital Gazette, Knowledge, Literary Digest.

### Scraps and Gleanings.

The new asylum for the city of Glasgow at Gartloch is now in working order.

The proposal to erect a cottage hospital at Sleaford has fallen through.

The Birmingham Children's Hospital has recently been the recipient of two legacies of £1,000 each.

The proposed new wing to be added to the Leith Hospital will raise the accommodation from 74 to 100 beds.

The Governors of the Victoria Hospital at Folkestone have decided to admit the Press to their quarterly meetings.

The receipts for the year 1896 at the Victoria Cottage Hospital, Romford, amounted to £334 and the expenditure to £344.

The centenary of the Sheffield Royal Infirmary takes place in October next. The infirmary was opened on October 4th, 1797.

The "life" of recommendations for both in and out patients at the Sheffield Royal Infirmary has been cut down to six weeks.

Some 30 mills in Oldham now make periodical collections on behalf of the Hospital Saturday Fund, in place of one annual collection.

The Government clerks in Ceylon, it is said, propose to present the General Hospital at Colombo with a new ward as a memorial of the Jubilee.

The Victorian Convalescent Home for Women is to be opened temporarily at Bognor for women resident within the administrative county of Surrey.

Out of the £4 per head which it is calculated the in-patients cost last year at the Wimbledon Cottage Hospital, it is noteworthy that the patients themelves contributed £1.

The number of patients admitted to the wards of the Retford Cottage Hospital show a steady increase year after year. In 1894-5 they numbered 79; in 1895-6, 104; and in 1896-7, 122.

One thousand and thirty-eight patients in 1896 passed through the Seaside Convalescent Hospital at Seaford, which was one of the first convalescent institutions established at the seaside.

The patients admitted to the wards of the Arbroath Infirmary during the year ended June 30th last numbered 160. Three hundred and twenty cases were, in addition, treated in the dispensary.

One hundred and ninety in and 1,710 out-patients received treatment at the Teignmouth Infirmary during the past year. The receipts amounted to £835, and the expenditure to £920, leaving a deficiency of £85.

At the recent annual meeting of the Sheffield Royal Infirmary the rules were altered so as to provide for an election committee to fill the vacancies on the honorary medical staff, thus doing away with the system of canvassing which had been in force up to that date.

The Stratton Cottage Hospital, Cornwall—one of the oldest in the kingdom—had in its wards during the past year 28 patients, or eight less than in 1895-6. The average stay was 43 days, against 30 days, and the average weekly cost of a patient was 23s. 0½d., compared with 19s. the previous year.

The number of patients treated at the Coombe Lying-in Hospital during the past year was: Labour wards, 544; chronic wards, 208; extension maternity department, 1,634; special dispensary, 2,186; and general dispensary, 10,341—a total of 1,616 more patients than were treated during the previous year. Not one death occurred in the intern midwifery department.

Nine less patients (59 against 68) were treated in the wards of the Wellington and District Cottage Hospital during the year ended May 31st last than were treated in 1895-6. The expenditure was slightly less—£357 against £384, but in consequence of a falling off in the receipts the accounts showed a debit balance of £5 odd. The average number of days each in-patient was resident was 41¼.

The challenge shield, value 200 guineas, offered for competition by the Volunteer Medical Association to encourage ambulance training in volunteer regiments was won this year by a team of four stretcher bearers from the 2nd V.B. Royal Fusiliers, who scored 252 marks out of a possible 300 in stretcher and waggon drill, and examinations in "first aid," bandaging, and anatomy and physiology.

Thanks to the energy of the ex-Mayor of Ipswich, the receipts of the East Suffolk and Ipswich Hospital for the year ended May 31st last show a decided improvement when compared with those of the previous year. Nearly every item shows an increase, annual subscriptions one of about £200, and the net result is that in place of a debit balance of nearly £900 there is a credit balance for the year of over £1,600.

It does not often happen that the opponents of an infectious diseases hospital scheme are willing to pay for their opposition. Sir William Cunliffe Brooks, however, who has associated himself with the people of Aboyne in objecting to the proposal to place a contagious diseases hospital near the town, has offered the district committee £1,000 in order to enable them to get rid of the scheme and to select a site elsewhere.

During the last ten or twelve years the following additions have been made to the West Kent Hospital at Maidstone: A casualty department, an operating theatre, a nurses' home, the Hallowes' Ward, and an isolation block for infectious cases. A new wing is about to be added so as to allow of the enlargement of the out-patients' department, which it has long been felt is too small for the requirements of the hospital.

Since January of this year nine new branches of the Children's Happy Evenings Association have been opened, viz.: Two at Deptford, one at Whitechapel, and two each at Wandsworth, Chelsea, and Bethnal Green. The objects of this association were happily summed up by the Duke of Portland at the annual meeting held recently, when he said that it aimed at making England the playground and Paradise of the poorest children.

During the year 1896 the Cabdrivers' Benevolent Association granted 60 annuities of £20 a-year each to aged and infirm cabdrivers, loaned without interest small sums to 91 members, made free grants to some 70 applicants, relieved 10 widows with temporary monthly pensions, and rendered legal assistance to 46 members. With reference to the latter we may mention that out of 16 summonses against members during the year 10 were dismissed

### The Student of Medicine.

### APPOINTMENTS.

N. S. Bickford, M.R.C.S.Eng., L.R.C.P.Lond., appointed House Surgeon to the Hospital for Sick Children, Great Ormond Street. W. Bulloch, M.D., appointed Bacteriologist to the Hospital and Lecturer on Bacteriology in the London Hospital Medical School. A. Dingwall, M.B., appointed Medical Officer of Health for the Kington Urban District and Medical Officer for the Pembridge District of the Kington Union. Dr. E. G. Dobbin, appointed Assistant Medical Officer at the St. John's Road Workhouse and Infirmary of the Islington Union. D. K. Draffin, L.R.C.P., L.R.C.S.I., appointed Medical Officer for the Troedyrhiw District of the Merthyr Union. C. F. Gross, M.R.C.S., L.R.C.P.Lond., appointed Medical Officer for the Seventh District of the East Ashford Union. R. M. Heggs, L.S.A.Lond., appointed Assistant House Surgeon to the Wolverhampton and Staffordshire General Hospital, Wolverhampton. A. O. Honnywill, L.R.C.S., L.R.C.P.Edin., appointed Medical Officer to the Epsom (Rural), Sutton, Carshalton, and Leatherhead Joint Isolation Hospital. C. S. Jago, M.R.C.S.Eng., L.S.A., appointed Medical Officer of Health for the St. Just Urban District. S. C. Jones, M.D.Lond., M.R.C.S.Eng., appointed Medical Officer for the Lower Town District of the Merthyr Union. W. R. Kingdon, M.B.Durh., B.S , appointed Resident Medical Officer of the Ticehurst Asylum, Sussex. H. A. Layton, L.R.C.P.Edin., M.R.C.S.Eng., appointed Medical Superintendent to the Bodmin Asylum. Dr. Long, appointed Medical Officer for the Second Chesham District of the Amersham Union. R. J. MacDermott, B.A.Dub., M.B., reappointed Medical Officer for the Northcapel District of the Petworth Union. J. Neil, M.D.-Aberd., appointed Superintendent and Secretary to the Warneford Asylum, Oxford. J. Palmer, L.R.C.P.Lond., M.R.C.S., appointed Medical Officer for the Snodland No. 5. District of the Malling Union. N. Raw, M.D.Durh., B.S., appointed Superintendent of the West Derby Union Infirmary, Mill Road, Liverpool. A. Scott, M.D.Glasg., appointed Certifying Factory Surgeon for the Glasgow District. D. Stenhouse, L.R.C.P., L.R.C.S.Edin., appointed Medical Officer for the Arnold District of the Basford Union. Dr. Stephens, appointed Medical Officer and Public Vaccinator of the West Auckland District of the Auckland Union. R. McClean Stokes, L.R.C.P., L.R.C.S.Edin., appointed Medical Officer for the Monmouth District of the Monmouth Union. S. R. Walker, M.R.C.S., L.R.C.P., appointed House Surgeon to the Westminster Hospital. F. K. Wilson, M.B., B.S.Lond., M.R.C.S.Eng., L.R.C.P.Lond., appointed House Surgeon to the Westminster Hospital. C. G. Hoysted, L.R.C.P., L R.C.S.Edin., appointed Medical Officer and Public Vaccinator for the Third District of the Dartford Union. J. Lloyd, M.B., F.R.C.S., appointed Honorary Consulting Surgeon to the West Bromwich District Hospital. A. Macfadyen, M.D.Edin., appointed Director of the British Institute of Preventive Medicine. J. Maclaren, M.D.Edin., appointed Certifying Factory Surgeon for the Oughtibridge District, and Medical Officer and Public Vaccinator to No. 3 District of the Wortley Union. F. P. Monckton, L.R.C.P., L.R.C.S., appointed Assistant Medical Officer at the Roxburgh District Asylum, Melrose. J. A. Morgan, M.R.C.S., L.R.C.P., appointed Medical Officer for the Llanddulas and Llanwrtyd District of the Builth Union. W. H. Rowell, M.B., B.S.Durh., appointed Junior House Physician, Royal Infirmary, Newcastle-on Tyne.

### ROYAL COLLEGE OF PHYSICIANS AND SURGEONS, EDINBURGH.

The following are some of the questions set in the final examination in July for the triple qualification :—

Medical Jurisprudence and Hygiene (Three questions only to be answered. The Candidate may answer either No. 1 or No. 2, but must answer Nos. 3 and 4).

1. What is the distinction between death by hanging, death by strangulation, and death by suffocation? To which of these would you refer death by overlaying? What lines of defence might be adopted with regard to this kind of death, and how could these be met in a court of law?

2. A dead child is found with its skull fractured. What appearances would enable you to certify that the injury had been caused during delivery?

3. What are the symptoms and appropriate treatment of carbolic acid poisoning? Mention two distinctive tests.

4 (Hygiene.) To what causes may an outbreak of typhoid be attributed? What is the duty of the health officer in each of the special circumstances?

